# Attenuation of the programmed cell death-1 pathway increases the M1 polarization of macrophages induced by zymosan

**DOI:** 10.1038/cddis.2016.33

**Published:** 2016-02-25

**Authors:** W Chen, J Wang, L Jia, J Liu, Y Tian

**Affiliations:** 1 Department of Medical Science of Laboratory, Liaoning University of Traditional Chinese Medicine, Shenyang, China; 2 Key Laboratory of Ministry of Education for TCM Viscera-State Theory and Applications, Liaoning University of Traditional Chinese Medicine, Shenyang, China; 3 Graduate Institute, Liaoning University of Traditional Chinese Medicine, Shenyang, China; 4 College of Animal Science and Veterinary Medicine, Shenyang Agricultural University, Shenyang, China; 5 Medical College, University of South China, Hengyang, China

## Abstract

Programmed cell death-1 (PD-1) is a member of the CD28 superfamily that delivers negative signals on interaction with its 2 ligands, PD-L1 and PD-L2. We assessed the contribution of the PD-1 pathway to regulating the polarization of macrophages that promote inflammation induced by zymosan. We found that PD-1^−/−^ mice developed robust peritonitis with more abundant infiltration of M1 macrophages, accompanied by higher levels of pro-inflammation factors, especially monocyte chemotactic protein-1 (MCP-1) compared with wild-type controls *ex vivo* and *in vitro*. Our results indicated that PD-1 deficiency promotes M1 rather than M2 polarization of macrophages by enhancing the expression of p-STAT1/p-NF-*κ*B p65 and downregulating p-STAT6. We found that PD-1 engagement followed by zymosan stimulation might primarily attenuate the phosphorylation of tyrosine residue in PD-1 receptor/ligand and the recruitment of SHP-2 to PD-1 receptor/ligand, leading to the reduction of M1 type cytokine production.

Macrophages display highly dynamic phenotypes that react to their microenvironment, with classically activated, pro-inflammatory macrophages and anti-inflammatory macrophages designated as M1 (classical macrophages) and M2 macrophages (alternative macrophages).^1^ M1 macrophages characteristically generate high levels of IL-12, monocyte chemotactic protein-1 (MCP-1) and low amounts of IL-10 and express high amounts of the chemokine receptor CCR7 (CD197). Functionally, M1 macrophages are highly inflammatory and effective killer cells for microorganisms and tumor cells. M2 macrophages, on the other hand, are induced by a variety of stimuli, such as IL-4 or IL-13 (M2a), immune complexes (M2b), IL-10 and glucocorticoids (M2c).^2^ They all exhibit low levels of IL-12 and express high levels of IL-10, mannose receptors (CD206) and scavenger receptors (CD163). M2 macrophages are modulators of inflammatory responses. The peritoneal cavity is a unique compartment within which a variety of immune cells reside, and from which macrophages are commonly drawn for functional studies. The existence of two resident macrophage subsets coexisting in peritoneal cavity was described recently.^3^ One was CD11b^high^ F4/80^high^, called the large peritoneal macrophages (LPMs), which were the main population of macrophages in unstimulated animals but disappear rapidly from peritoneal cavity following lipopolysaccharide or thioglycollate stimulation. The other subset was CD11b^int^ F4/80^int^ MHC II ^high^, referred to as small peritoneal macrophages (SPMs). SPMs, which predominate in peritoneal cavity after lipopolysaccharide or thioglycollate stimulation, derive from blood monocytes that rapidly enter the peritoneal cavity after stimulation and differentiate to mature SPMs within 2–4 days. SPMs appear to develop an efficient profile to control infections as M1 macrophages, whereas LPMs assume a role in the maintenance of peritoneal cavity physiological conditions as M2 macrophages.^4^

Programmed cell death-1 (PD-1) is a well-characterized receptor on T cells for PD-L1 and PD-L2. PD-1 is an activation antigen which is upregulated by T-cell activation and returns to basal levels following antigen clearance. The PD-1 molecule has been recognized as a hallmark of T-cell exhaustion, and PD-1-expressing antigen-specific T cells are dysfunctional in cytokine production and proliferation on antigen restimulation in a variety of viral infections.^5^ Anti-PD-1 antibody therapies are being developed to enhance T-cell responses in patients with cancers or chronic viral infections, such as HIV and hepatitis C.^6^ Although the role of PD-1 in adaptive immunity has been well characterized,^7, 8^ its function in innate immunity has seldom been investigated. Whether PD-1 has an important role on the polarization of macrophages/monocytes in peritoneal cavity to exert significant anti-inflammatory and atheroprotective effects has not been studied previously. In this study, we found that PD-1 deficiency promoted M1 polarization of macrophages/monocytes and exacerbated inflammation induced by zymosan via enhancing the phosphorylation of STAT1/p-NF-*κ*B p65. We demonstrated that PD-1 engagement followed by zymosan stimulation might primarily attenuate the phosphorylation of tyrosine residue in PD-1 receptor and the recruitment of SHP-2 to PD-1 receptor, leading to the reduction of M1 type cytokine production.

## Results

### The subsets of peritoneal cells in the peritoneal cavity by flow cytometry

**Figure 1:**
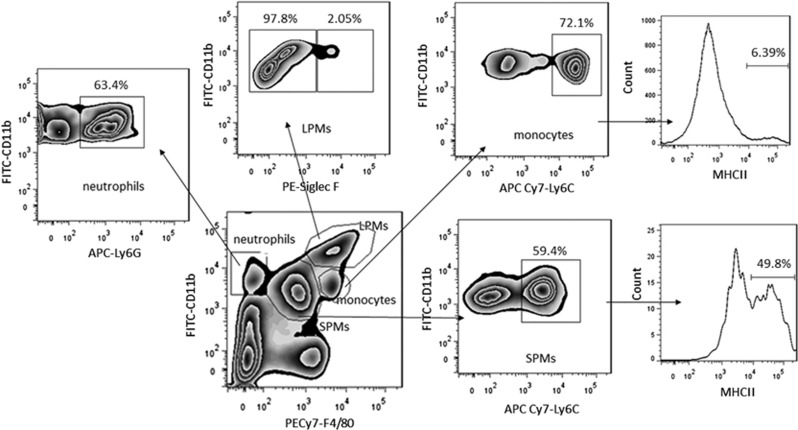
Gating of macrophages/monocytes/neutrophils subsets in peritoneal exudates. Cells collected from the peritoneal cavity were stained with appropriate anti-mouse mAbs, and analyzed by flow cytometry. It showed four subsets in the peritoneal cells gated in CD19^−^ population: CD11b^high^ F4/80^high^ CD11c^low^ in large size (LPMs), CD11b^int^ F4/80^int^ LY6C^int^ CD11c^−/low^ MHC II ^high^ in small size (SPMs), CD11b^int^ LY6C^high^ MHC II ^low^ CD11c^−^^/low^ (monocytes), CD11b^int^ F4/80^−^ LY6G^high^ (neutrophils)

Live cells were gated by violet^−^ population, and then we defined four subsets in the cells gated in CD19^−^ population:^9^ CD11b^high^ F4/80^high^ CD11c^low^ in large size (LPMs), CD11b^int^ F4/80^int^ LY6C^int^ CD11c^−^^/low^ MHC II ^high^ in small size (SPMs), CD11b^int^ LY6C^high^ MHC II ^low^ CD11c^−^^/low^ (monocytes), CD11b^int^ F4/80^−^ LY6G^high^ (neutrophils), as shown in Figure 1. By maintaining a high F4/80 cutoff combined with FSC/SSC and pulse-width gating, we tried to ensure minimal contamination with eosinophils or peritoneal dendritic cell-like cells. Eosinophils were explicitly excluded by the gating with CD11b^int^ Siglec-F ^high^ from the SPMs and LPMs compartments.^10^

**Figure 2:**
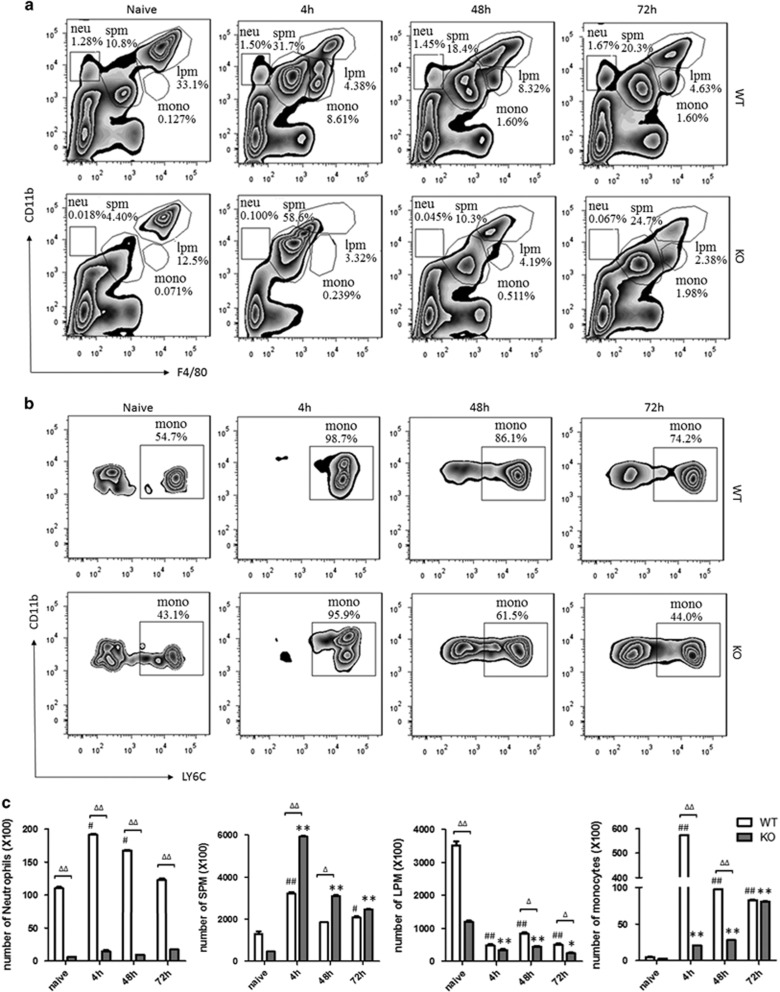
The subsets of macrophages/monocytes/neutrophils in peritoneal exudates. The percentage of LPMs, SPMs, monocytes and neutrophils in the peritoneal exudates from PD-1^−/−^ (KO) mice were analyzed by flow cytometry compared with that in WT mice after the injection of zymosan for 4, 48 and 72 h. (**a**) LPMs, SPMs, monocytes and neutrophils were shown with gate of CD11b+ and/or F4/80+. (**b**) SPMs and monocytes were shown with gate of CD11b+ and/or LY6C+. (**c**) the numbers of LPMs, SPMs, monocytes and neutrophils in peritoneal exudates. *n*=3 for each group. Mean±S.D., ^#^*P*<0.05 *versus* WT naive, ^##^*P*<0.01 *versus* WT naive,*P<0.05 *versus* KO naive, **P<0.01 *versus* KO naive, ^Δ^*P*<0.05 WT *versus* KO, ^ΔΔ^*P*<0.01 WT *versus* KO

In WT mice, LPMs were the predominant cells (35.5±4.2%) which first decreased after inflammation initiation (4.6±1.4% at 4 h), as the physiologic response already known as ‘macrophage disappearance reaction',^11^ then LPM frequencies dwindled to less than 10% after 48 h stimulation (8.4±1.8%), and were kept at a low level until 72 h (5.3±1.6%); SPMs became the dominant subset in the peritoneal cavity (36.0±3.4%) at 4 h and decreased at 48 and 72 h, but SPMs were the main population during the whole phase after simulation. Monocytes infiltrated the peritoneal cavity 4 h after injection (9.32±2.3%), and then decreased at 48 h (1.7±0.3%) and 72 h (1.6±0.4%). We found that it was not obvious that neutrophils trafficking into the inflamed peritoneum, although the absolute number cells were increased at 4 h, perhaps the percentage of neutrophils contained in the total cells were too low (Figures 2a–c). Compared with WT mice, in PD-1^−/−^ mice, LPMs were lower in PD-1^−/−^ naive mice (13.5±2.6%), and they decreased after 4 h (3.6±1.1%), then kept at a low level until 72 h; SPMs infiltrated promptly into the peritoneal cavity at 4 h (56.0±5.3%) and became the predominant subset, and then they decreased at 48 and 72 h. The percentage of SPMs was kept at a higher level during the whole phase after zymosan stimulation than that in WT mice; the infiltration of monocytes was delayed until 48 h (0.7±0.2%) after injection, and increased gradually at 72 h (1.6±0.4%); interestingly, we did not find infiltration of neutrophils in KO mice.

### Intracellular staining of cytokines in peritoneal macrophages

**Figure 3:**
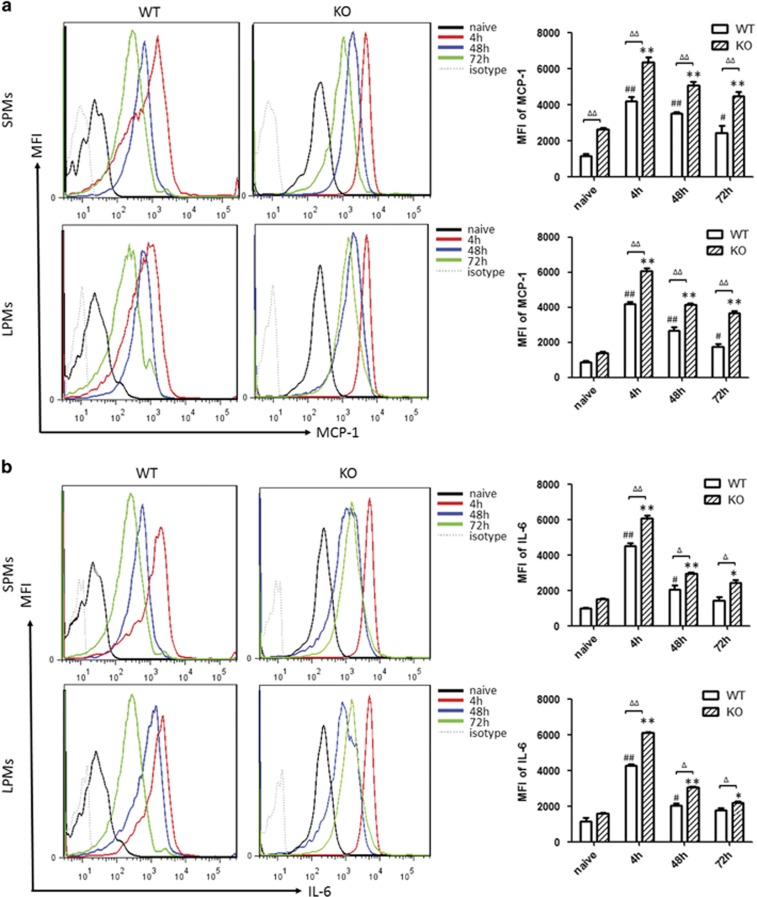
The intracellular staining of cytokines in peritoneal macrophages. Cells were collected from peritoneal cavity after zymosan induction for 4, 48 and 72 h separately. Cells were stained with membrane markers (CD11b-FITC, F4/80-PECy7, CD11c-PerCP-Cy5.5, CD19-BV650), then permed with perming buffer, and staining of intracellular cytokines was continued. The mean fluorescence intensity (MFI) of MCP-1 (**a**) and IL-6 (**b**) were analyzed in the SPMs and LPMs by gates as previously. *n*=3 for each group. Mean±S.D., ^#^*P*<0.05 *versus* WT naive, ^##^*P*<0.01 *versus* WT naive,*P<0.05 *versus* KO naive, **P<0.01 *versus* KO naive, ^Δ^*P*<0.05 WT *versus* KO, ^ΔΔ^*P*<0.01 WT *versus* KO

Cells from the peritoneal cavity were collected after zymosan induction for 4, 48 and 72 h separately. Cells were stained with live/dead and membrane markers and then staining of intracellular cytokines was continued. The mean fluorescence intensity of IL-6 and MCP-1 were analyzed in the SPMs and LPMs by gates as previously. The results showed that SPMs produced higher levels MCP-1 and IL-6 compared with LPMs in all three time points. It means that SPMs expressed more pro-inflammatory phenotype than LPMs. The mean fluorescence intensity of MCP-1 and IL-6 were increased quickly in both SPMs and LPMs from WT mice and PD-1^−/−^ mice at 4 h after induction by zymosan compared with naive mice (*P*<0.01). MCP-1 and IL-6 in both SPMs and LPMs from PD-1^−/−^ mice showed obviously higher levels compared with WT mice (*P*<0.01) in Figures 3a and b. The levels of MCP-1 and IL-6 were decreased after induction by zymosan in both SPMs and LPMs from both PD-1^−/−^ mice and WT mice at 48 and 72 h, compared with 4 h separately. For the production of MCP-1 and IL-6, there were obvious differences between KO mice and WT mice in all three time points (*P*<0.01 or 0.05). The level of IL-6 was not significantly increased at 72 h in both SPMs and LPMs from WT mice compared with naive mice (*P*>0.05), shown in Figure 3b. These results showed an increasing tendency in the expression of the pro-inflammatory cytokine in PD-1^−/−^ mice.

### The analysis of cytokines in peritoneal cavity by ELISA

**Figure 4:**
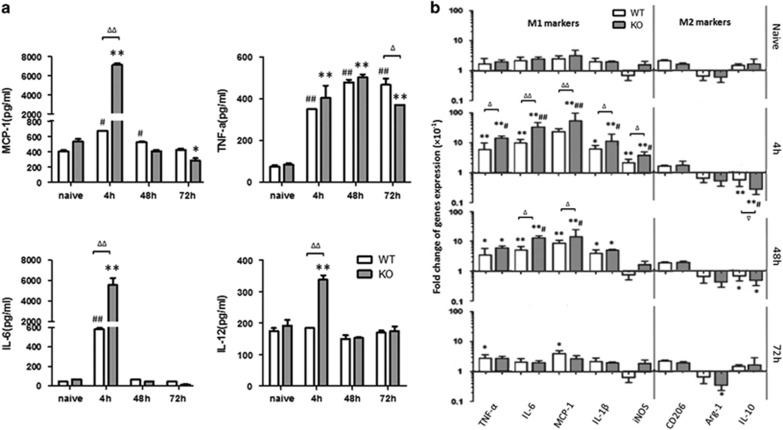
Cytokine expression in the peritoneal lavage and macrophages. (**a**) The levels of cytokines in the peritoneal lavage. The levels of MCP-1, TNF-*α*, IL-6 and IL-12 in the peritoneal exudates from WT mice or PD-1^−/−^ (KO) mice were detected by ELISA at 4, 48 and 72 h after zymosan injection. (**b**) The expression of relative mRNA of M1/M2 markers in the peritoneal macrophages. The expression of genes of cytokines (TNF-*α*, IL-6, MCP-1, IL-1*β*, iNOS, CD206, Arg-1 and IL-10) in the peritoneal macrophages from WT mice or PD-1^−/−^ (KO) mice were sorted by flow cytometry in gate of CD11b^+^F4/80^+^ after zymosan injection for 4, 48 and 72 h, and then analyzed by RT-qPCR. *n*=3 for each group. Mean±S.D., ^#^*P*<0.05 *versus* WT naive, ^##^*P*<0.01 *versus* WT naive,*P<0.05 *versus* KO naive, **P<0.01 *versus* KO naive, ^Δ^ *P*<0.05 WT *versus* KO, ^ΔΔ^ *P*<0.01 WT *versus* KO

We performed ELISA for cytokines in the peritoneal exudates at three time points after zymosan injection. As shown in Figure 4a, the levels of MCP-1, IL-6 and IL-12 were increased quickly in PD-1^−/−^ mice at 4 h, and the difference was significant compared with WT mice (*P*<0.01), whereas the levels of MCP-1, IL-6 and IL-12 were shown to have no obvious difference between KO mice and WT mice at 48 and 72 h (*P*>0.05). For the production of TNF-*α*, there were no obvious differences between KO mice and WT mice in all three time points (*P*>0.05), although the levels of TNF-*α* were significantly increased at 4 h in both KO mice and WT mice compared with naive mice (*P*<0.01), as shown in Figure 4a. These results showed an increasing tendency in the expression of the pro-inflammatory cytokine in PD-1^−/−^ mice.

### The analysis of genes expression in peritoneal macrophages by RT-qPCR

We performed RT-qPCR for genes of cytokines (TNF-*α*, IL-6, MCP-1, IL-1*β*, iNOS, CD206, Arg-1 and IL-10) on harvested three time points after zymosan injection. As shown in Figure 4b, there was a significant increase of pro-inflammatory cytokines (TNF-*α*, IL-6, MCP-1, IL-*β*, iNOS) in the PD-1^−/−^ mice from 4 h after the injection of zymosan until 48 h, compared with WT mice (*P*<0.01 or 0.05), whereas a decreasing tendency in the expression of the anti-inflammatory cytokine (CD206, Arg-1 and IL-10) was observed (*P*<0.01 or 0.05). The expression of the cytokine mRNA almost returned to the basal levels 72 h after the treatment compared with naive mice (*P*>0.05).

### The expression of PD-1 from BMDMs induced by zymosan

**Figure 5:**
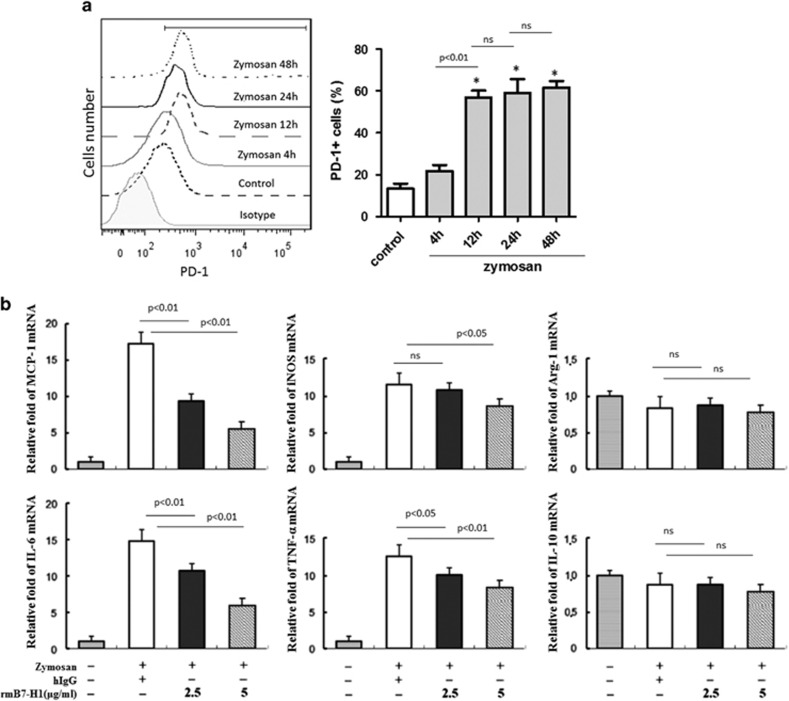
The expression of PD-1 and cytokine mRNA in BMDMs induced by zymosan. (**a**) The expression of PD-1 on BMDMs induced by zymosan. BMDMs from WT mice were activated with zymosan for 4, 12, 24 and 48 h separately to stimulate TLR2. Cells were stained with PE-conjugated anti-mouse PD-1 antibody, and analyzed by flow cytometry. Data are presented as the percentage of positive cells. *n*=3 for each group. Mean±S.D., *P<0.01 *versus* untreated control. (**b**) The levels of cytokine genes in BMDMs induced by PD-L1 in the presence of zymosan. BMDMs from WT mice were pretreated with the indicated concentrations of rmB7-H1 or hIgG for 2 h and stimulated with 20 *μ*g/ml zymosan for 12 h. RT-PCR was performed to analyze iNOS, IL-6, TNF-*α*, MCP-1, Arg-1 and IL-10 gene transcription

To assess the effect of PD-1 signaling on immune-regulated gene expression in BMDMs, we first examined PD-1 expression on BMDMs after activation by zymosan for 4, 12, 24 and 48 h using flow cytometry. Flow cytometric analysis showed that PD-1 expression was detected in unstimulated BMDMs, and upregulated in response to zymosan for 12 h. Specifically, PD-1 level was about four times higher after stimulation by zymosan for 12 h than that of unstimulated cells. But about the upregulation of PD-1 on zymosan-stimulated BMDMs, there was no significant difference in 12 h, compared with 24 or 48 h (Figure 5a). Thus, we chose 12 h as the timepoint to perform the subsequent experiment.

### The levels of cytokine genes in BMDMs induced by PD-L1 in the presence of zymosan

We examined the effects of PD-1 on the polarization of primary BMDMs isolated from WT mice. The expression of genes involved in immune regulation in BMDMs were detected after treatment with recombinant mouse B7-H1.Fc (rmB7-H1) in the presence of zymosan. As shown in Figure 5b, PD-1 engagement with rmB7-H1 inhibited the expression of cytokine genes including iNOS, IL-6, TNF-*α* and MCP-1, but not Arg-1 and IL-10. Especially, the expression of mRNAs of MCP-1 and IL-6 were downregulated significantly compared with cells only stimulated with zymosan. These results indicated that the activation of PD-1 pathway could shift macrophage polarization toward the M2 phenotype instead of the M1 phenotype. As MCP-1 is a key cytokine in the development of peritonitis, we focused on the regulation of MCP-1 gene expression in BMDMs treated with rmB7-H1 in the following experiments.

### The expression of MCP-1 in BMDMs induced by zymosan mediated by PD-1-specific signaling

**Figure 6:**
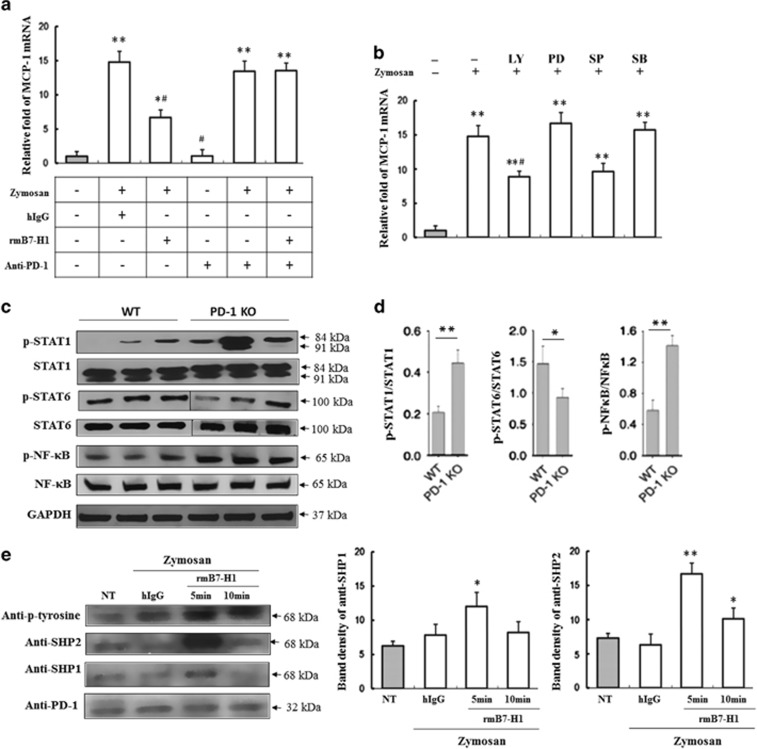
(**a**) The expression of MCP-1 mRNA in BMDMs induced by zymosan via PD-1-specific signaling. MCP-1 mRNA in zymosan-stimulated BMDMs after PD-1 was stimulated with/without antagonistic anti-PD-1 mAb. (**b**) Effect of PD-1 engagement on signaling pathway responsible for MCP-1 gene expression. BMDMs were pretreated with the indicated inhibitors for 30 min and stimulated with 20 *μ*g/ml zymosan for an additional 12 h in the presence or absence of various signaling inhibitors. Total RNA was prepared and analyzed by RT-qPCR for mouse MCP-1 or GAPDH. LY (PI3K inhibitor); PD (ERK inhibitor); SP (JNK inhibitor); SB (p38 inhibitor). *n*=3 for each group. Mean±S.D., *P<0.05 *versus* untreated control, **P<0.01 *versus* untreated control, #*P*<0.01 *versus* zymosan-treated group. (**c**) PD-1 deficiency influences the polarization of peritoneal macrophages via signal transducer and activator of transcription1 (STAT1), nuclear factor kappa-B (NF-*κ*B) and STAT6. Western immunoblot for phosphorylated p-STAT1, STAT1, p-NF-*κ*B, NF-*κ*B, p-STAT6 and STAT6 in macrophages stimulated with zymosan injection for 4 h, *n*=3. (**d**) Densitometry analysis for p-STAT1/STAT1, p-NF-*κ*B/ NF-*κ*B and p-STAT6/ STAT6 relative to *β*-actin. *n*=3 for each group. Mean±S.D. (**e**) BMDMs were cultured with 20 *μ*g/ml zymosan for 12 h and stimulated with 5 *μ*g/ml rmB7-H1 at the indicated time. Cells were lysed in lysis buffer. The cell lysates were immunoprecipitated overnight with 5 *μ*g/ml of anti-mPD-1, and protein A/G-agarose was used for precipitation. The suspended pellets were subjected to western blotting with anti-PD-1, anti-phosphotyrosine, anti-SHP-2 and anti-SHP-1 antibodies. *n*=3 for each group. Mean±S.D., *P<0.05 *versus* untreated control, **P<0.01 *versus* untreated control

In order to confirm whether the inhibition of MCP-1 expression is mediated by PD-1-specific signaling, we made further investigation on MCP-1 production in zymosan-stimulated BMDMs using antagonistic anti-PD-1 mAb (clone J43), which is reported to block B7-H1.Fc binding to PD-1.^11^ As expected, the blockade of PD-1 engagement with anti-PD-1 mAb restored MCP-1 gene expression as revealed by RT-PCR (Figure 6a). These results indicated that PD-1 delivered a negative signal into zymosan-stimulated BMDMs, leading to the suppression of MCP-1 production. It has been reported that MCP-1 production is mediated by activation of c-Jun N-terminal kinase, the activation protein-1 (AP-1) and NF-*κ*B transcription factors.^12, 13, 14^ Our results showed that SP600125 (a JNK inhibitor) and LY294002 (a PI3K/AKT inhibitor), but not PD98059 (a MEK 1/2 inhibitor) and SB203580 (a p38 inhibitor), strongly suppressed MCP-1 gene transcription in zymosan-stimulated BMDMs (Figure 6b). These results suggest that JNK and PI3K/AKT signaling pathways are involved in MCP-1 induction in macrophages in response to zymosan. There was no significant difference in phosphorylation of other kinase such as ERK in zymosan-stimulated BMDMs with or without rmB7-H1.

### The signal pathway regulated PD-1-mediated suppression of MCP-1 production

STAT1 and NF-*κ*B are two important transcription factors that regulate the expression of genes characteristic of the M1 phenotype. In contrast, IL-4 and IL-13 promote the M2 phenotype via STAT6 activation.^15, 16^ Because STAT1 and STAT6 have a reciprocal inhibitory relationship in macrophage polarization, our next aim was to determine whether PD-1 regulates M1 and M2 polarization through the STAT1/NF-*κ*B or STAT6 pathways, respectively. In our study, the levels of p-STAT1 and p-NF-*κ*B p65 were significantly increased in peritoneal macrophages from PD-1 KO with zymosan stimulation compared with cells from WT mice. In contrast, the level of p-STAT6 was significantly decreased (Figures 6c and d). Our results indicate that PD-1 deficiency promotes M1 rather than M2 polarization of macrophages by enhancing the expression of p-STAT1/p-NF-*κ*B p65 and downregulating p-STAT6.

It is known that MCP-1 (and most chemokines) are driven by downstream of TLR2. We detected the protein expression of PD-1/PD-L1 pathway to confirm whether PD-1 deficiency contributed to the reduction of MCP-1 after zymosan stimulation. As previous studies^17, 18^ reported that PD-1 engagement by PD-L1 induced the phosphorylation of tyrosine residue from immune-receptor tyrosine-based inhibitory motif (ITIM) in cytoplasmic domain, recruiting SHP-2 in B cells, we investigated whether PD-1 engagement could impact on the phosphorylation of PD-1 and SHP-1 and SHP-2 recruitment in BMDMs. When BMDMs were stimulated with rmB7-H1, PD-1 was phosphorylated at an early time compared with the hIgG-treated cells. Furthermore, PD-1 engagement with rmB7-H1 actively recruited SHP-2 to the PD-1 cytoplasmic tail but weakly recruited SHP-1 (Figure 6e). The result showed that PD-1 signaling might primarily attenuate the phosphorylation of tyrosine residue in PD-1 receptor/ligand and the recruitment of SHP-2 to PD-1 receptor/ligand, leading to the reduction of M1 type cytokine production.

## Discussion

We found that the macrophage/monocyte population after zymosan stimulation shifted with time toward expression of the SPM phenotype. SPMs infiltrated into the peritoneal cavity promptly at 4 h and then became the dominant subset in the peritoneal cavity during the whole phase after zymosan simulation in both WT mice and PD-1^−/−^ mice. The percentage of SPMs kept higher during the whole phase after zymosan stimulation in PD-1^−/−^ mice than that in WT mice; the infiltration of monocytes delayed in PD-1^−/−^ mice until 48 h after injection compared with 4 h in WT mice. Someone has previously reported flow cytometric phenotype of SPMs as a mixture with characteristics of inflammatory monocytes merging into inflammatory macrophage.^19^ The previous results clearly indicated that inflammation induced by zymosan stimulation induced the infiltration of Ly-6C^hi^ inflammatory blood monocytes that differentiated to SPMs that were phenotypically indistinguishable (currently) from typical resident SPMs in the peritoneal cavity from unstimulated mice.^20^ Thus, it indicated that a wave of blood monocytes entered the peritoneal cavity shortly after zymosan stimulation and, over time, differentiated to SPMs. It is interesting that we did not find infiltration of neutrophils in PD-1^−/−^ mice. It remains unclear whether PD-1 deficiency could influence the release of chemokines which could induce the infiltration of neutrophils. We would like to carry out some research on this direction in our future study.

We found that zymosan stimulated high level of pro-inflammatory cytokines production (especially accompanied with extreme high concentration of MCP-1) by the cells in peritoneal cavity of which SPMs were the predominant subset in PD-1^−/−^ mice. In contrast, the expression of anti-inflammatory cytokine showed some decreasing tendency. Thus, we think perhaps MCP-1 production was induced by zymosan resulting in the rapid entry of a sizable population of blood monocytes into the peritoneal cavity and these cells gradually differentiated into macrophages that express the typical M1 phenotype. This developmental process appears to continually be occurring at high levels MCP-1. However, without the severe disruption of the peritoneal economy by zymosan stimulation, it would have been difficult to detect this apparently continuous developmental process. As we know, MCP-1 is defined as one of the pro-inflammatory cytokine and it is a typical maker as M1 macrophages.^3^ Then we wanted to reveal whether PD-1 deficiency could influence the phenotype of macrophages in peritonitis induced by zymosan. However, owing to the distinctive responses mounted by LPMs and SPMs to zymosan stimulation, it made it difficult to map LPMs and SPMs into the M1/M2 model. Hence, we detected the mRNA expression of cytokines including both pro-inflammatory and anti-inflammatory cytokines in macrophages/monocytes that were collected from peritoneal cavity after zymosan injection. We found that there was a significant increase of the pro-inflammatory cytokine (TNF-*α*, IL-6, MCP-1, IL-*β*, iNOS) in the PD-1^−/−^ mice from 4 h after the injection of zymosan until 48 h, compared with WT mice, whereas a decreasing tendency in the expression of the anti-inflammatory cytokine (CD206, Arg-1 and IL-10) was observed. These results uncovered that PD-1 deficiency increased M1 polarization in peritonitis induced by zymosan.

MCP-1 is one of the key chemokines that regulates the migration and infiltration of monocytes/macrophages, and it has been demonstrated that MCP-1 could recruit monocytes into foci of active inflammation as a pro-inflammatory cytokine.^21^ It is produced by many cell types, including endothelial, fibroblasts, epithelial, smooth muscle, mesangial, astrocytic, monocytic and microglial cells.^22^ The data in our study showed that monocytes infiltrated the peritoneal cavity 4 h after injection and then decreased at 48 and 72 h. A previous study showed that a variety of bacterial-derived TLR ligands could induce PD-1 expression on human macrophages, suggesting that TLR signaling pathways played an important role in regulating PD-1.^23^ Although no ‘exhausted'-like phenotype has been observed in PD-1^+^ macrophages, the induced anti-inflammatory cytokine profile in PD-1^+^ macrophages has ramifications for the proper functioning of the immune system during infection.^24^

For mechanistic studies, we switched peritoneal macrophages to BMDMs for our following experiment, because *in vitro*, macrophages are usually stimulated with a single stimulus, which is only a limited amount of the wide variety of cytokines they may be exposed to in their *in vivo* microenvironment. Thus, M1 and M2 phenotypes represent simplified extremes of a ‘polarization spectrum', but *in vivo*, intermediate phenotypes are likely to exist. In addition, owing to the relatively low yields of macrophages that can be recovered from a mouse peritoneal cavity in steady-state conditions, intra-peritoneal injection of macrophage-eliciting agents to recruit immature macrophages is generally used to substantially increase macrophage numbers. Injection of thioglycollate broth for instance, recruits larger number of macrophages. However, it is controversial whether these macrophages are activated or not because their physiological characteristics may be different from resident cells.^3^ So BMDMs should be better for mechanistic studies than peritoneal macrophages.

Zymosan-activated macrophages are often classified as M1 macrophages owing to the increased levels of iNOS and IL-12 via TLR2 activation leads to NF-*κ*B activation.^25^ SHP-1 recruits the phosphorylation of ITIM domains on inhibitory receptors. Studies have shown that SHP-1 negatively regulates TLR-mediated production of pro-inflammatory cytokines by inhibiting the activation of NF-*κ*B and mitogen-activated protein kinase.^26^ NF-*κ*B and activating protein-1 (AP-1) are well known as pivotal transcription factors that regulate the expression of numerous genes during the activation of macrophages.^27, 28, 29^ In this study, we found that PD-1 deficiency enhanced the phosphorylation of STAT1/p-NF-*κ*B p65 and downregulated the phosphorylation of STAT6 to promote M1 rather than M2 polarization of macrophages. Our results showed that PD-1 engagement followed by zymosan stimulation might primarily attenuate the phosphorylation of tyrosine residue in PD-1 receptor/ligand and the recruitment of SHP-2 to PD-1 receptor/ligand, leading to the reduction of M1 type cytokine production. The results in our study indicated that impairment of the PD-1 pathway could boost the M1 polarization of macrophages induced by TLR2 ligand zymosan. This indicates that PD-1 blockade could help cure immune-regulated diseases such as cancer and in contrast, PD-1 agonist could had a vital role in anti-inflammatory in infectious disease such as arteriosclerosis.

## Materials and Methods

### Reagents and antibodies

Zymosan A from *Saccharomyces cerevisiae* was purchased from Sigma–Aldrich (St. Louis, MO, USA). Anti-mouse mAbs, such as CD11b-FITC, F4/80-PECy7, CD11c-PerCP-Cy5.5, CD19-BV650, MHC II (I-A/I-E)-PECy5, Siglec-F-PE, LY6G-APC, LY6C-APC-CY7, IL-6-PE and MCP-1-APC were purchased from eBioscience (San Diego, CA, USA). Recombinant mouse B7-H1/Fc chimera (1019-B7) was purchased from R&D Systems (Minneapolis, MN, USA). Human IgG for control experiments was purchased from Sigma–Aldrich. Anti-mouse PD-1 (clone J43) were purchased from eBioscience. LY294002, PD98059, SP600125 and SB203580 were purchased from Santa Cruz Biotechnology (San Diego, CA, USA). The following mAbs were used for western blotting and immunoprecipitation: anti mPD-1 (RPMI-30; eBioscience), anti-phosphotyrosine Ab (Cell Signaling Technology, Beverly, MA, USA), anti-SHP-1 (sc-287), anti-SHP-2 (sc-280), p-STAT1, STAT1, p-STAT6 and STAT6 (Cell Signaling Technology), anti-mouse IgG-HRP, anti-rabbit IgG-HRP Ab (Santa Cruz Biotechnology, Santa Cruz, CA, USA).

### Zymosan A preparation

Zymosan A from *Saccharomyces cerevisiae* (Sigma–Aldrich) was suspended in Hank's balanced salt solution with magnesium and calcium (HBSS+/+ Sigma–Aldrich) at 400 *μ*g/ml working solution and then heated at 90 °C. After cooling to room temperature, zymosan A was washed several times with HBSS+/+ and finally stored at −20 °C.

### Animal maintenance and peritonitis models

PD-1^−/−^ male mice on a C57BL/6 background, derived by targeted mutation in C57BL/6 ES cells, which results in the deletion of the IgV domain as described.^30^ C57BL/6 WT male mice (6–12 weeks) were given water *ad libitum* and were maintained on a 12/12-h light/dark cycle under pathogen-free/viral antibody-free barrier facility in the animal research building according to institutional and National Institutes of Health guidelines. Mice were injected intraperitoneally with 0.5 ml of zymosan A (200 *μ*g/mouse), as described in a previous study.^9^ The peritoneal cavity was lavaged 4, 48 and 72 h after injection.

### Collection of the lavage fluid and isolation of peritoneal cells

Mice were injected with 5 ml ice-cold PBS/2 mM EDTA washing solution. Gently, the mouse abdomen was massaged to ensure cells were loosely adherent to the peritoneal wall or other organs and became suspended in the lavage fluid. A 19-G needle was used to extract the lavage fluid from the peritoneal cavity gently and slowly. The peritoneal fluid was transferred to a 15-ml centrifuge tube and kept on ice. Lavage fluid was centrifuged at 300 × *g* for 5 min. Peritoneal cells were collected for flow staining (2.5 million). The supernatant was collected for ELISA assay.

### Flow cytometry

Cell suspensions were stained with violet live/Dead cell stain kit (Life Technologies, Eugene, OR, USA) for the analysis of viability, and then washed and stained with appropriate anti-mouse mAbs CD11b-FITC, F4/80-PECy7, CD11c-PerCP-Cy5.5, CD19-BV650, MHC II (I-A/I-E)-PECy5, Siglec-F-PE, LY6G-APC and LY6C-APC-CY7 (all from eBioscience) for 30 min. For the expression of PD-1, bone marrow–derived macrophages (BMDMs) were collected from cultures and washed with FACS buffer. And then, the cells were stained with PE-conjugated anti-mouse PD-1 (clone J43). For the intracellular staining of macrophages from peritoneal cavity, cells were stained with violet (live/Dead) and membrane markers (CD11b-FITC, F4/80-PECy7, CD11c-PerCP-Cy5.5, CD19-BV650), and then permed with perming buffer (BD Biosciences, San Jose, CA, USA), continued with staining of intracellular cytokines (IL-6-PE and MCP-1-APC, both from eBioscience). Data were acquired on BD fluorescence-activated cell sorting LSR-II (BD Biosciences) and analyzed with Flowjo software (TreeStar, Ashland, OR, USA).

### Analysis of cytokines by ELISA

Peritoneal exudates were centrifuged at 300 × *g* for 5 min. Supernatants were collected and stored at −80 °C for analysis. Analysis was performed by allowing quantification of MCP-1, TNF-*α*, IL-6 and IL-12 using ELISA kits, according to the manufacturer's instructions.

### Real-time RT-PCR analysis for peritoneal macrophages

Macrophages/monocytes were sorted by flow cytometry in gate within CD11b^+^F4/80^+^. Total RNA was isolated from the macrophages/monocytes by Trizol reagent (Sigma–Aldrich) according to the suppliers' instructions. Real-time RT-PCR was performed on a CFX 100 (Bio-Rad Laboratories, Hercules, CA, USA) cycler using one step real-time RT-PCR SYBR Green detection kit (Qiagen, Hilden, Germany). The specific primers for amplifying genes was listed in Table 1. Each sample was run in duplicate and the mean value of each set of duplicates was normalized to mouse *β*-actin and used to calculate relative gene expression.

### The induction of BMDMs by zymosan *in vitro*

Primary BMDMs were prepared from femur bone marrow of C57BL/6 or PD-1^−/−^ mice as previously described.^31^ Briefly, bone marrow cells were isolated from mouse femurs and cultured for 7 days in DMEM with 10% FBS, 100 U/ml penicillin/streptomycin and 20% L-929 cell line-conditioned media, which contains cell line-produced M-CSF. Purity of BMDMs (>90%) was determined by flow cytometry within gate of CD11b^+^ CD11c^−^ MHC II^+^. Cells were treated with 20 *μ*g/ml zymosan (InvivoGen, San Diego, CA, USA),^9^ with/without 20* μ*M LY294002, 10 *μ*M PD98059, 10* μ*M SP600125 and 10 *μ*M SB203580 for 4 h. Then, the expression of MCP-1 mRNA was detected by real-time RT-PCR.

### Expression of PD-1 on BMDMs

BMDMs from C57BL/6 mice were stimulated by zymosan (20 *μ*g/ml) for 4, 12, 24 and 48 h separately, stained with PE-conjugated anti-mouse PD-1 antibody, and the expression of PD-1 on BMDMs were detected by flow cytometric analysis, compared with unstimulated cells (control) and isotype control.

### Levels of cytokines mRNA in BMDMs

BMDMs from WT C57BL/6 mice were pretreated with the indicated concentrations of rmB7-H1 or hIgG for 2 h and stimulated with 20 *μ*g/ml zymosan for 12 h. RT-PCR was performed to analyze iNOS, IL-6, TNF-*α*, MCP-1, Arg-1 and IL-10 gene transcription.

### The expression of MCP-1 mRNA in BMDMs induced by zymosan mediated by PD-1 specific signaling

In order to confirm whether the inhibition of MCP-1 expression is mediated by PD-1 specific signaling, we made further investigation on MCP-1 production in 20 *μ*g/ml zymosan-stimulated BMDMs using PD-1 with/without antagonistic anti-PD-1 mAb (clone J43) which was reported to block B7-H1.Fc binding to PD-1.^11^

### Western blot and immunoprecipitation

For immunoprecipitation, BMDMs stimulated with zymosan (20 *μ*g/ml) for 12 h were washed twice with cold PBS and resuspended in 0.5 ml of PBS. The zymosan-stimulated BMDMs were stimulated with rmB7-H1 at 37 °C for 5 and 15 min. Cells were lysed in 0.5 ml of 1% NP-40 lysis buffer for 30 min and pelleted at 14 000 r.p.m. for 10 min at 4 °C. To detect PD-1 phosphorylation and recruitment of SHP-1 and SHP-2, whole cell lysates were incubated overnight with 5 *μ*g/ml anti-mPD-1 (RPMI-30; eBioscience). The next day the samples were incubated with protein A/G-agarose (sc-6244; Santa Cruz Biotechnology) for an additional 3 h at 4 °C, then washed five times with 1% NP-40 lysis buffer and finally resuspended in SDS loading buffer. The immunoprecipitates were resolved by SDS-PAGE and transferred to nitrocellulose. Blots were probed with appropriate primary and secondary antibody combinations, and proteins were visualized by using an ECL kit (Pierce, Rockford, CA, USA).

### Statistical analysis

Differences between two groups were tested using the Mann–Whitney test. All analyses were performed with GraphPad Prism 5 software. For multiple comparisons, the *P* value was determined by ANOVA followed by a Bonferroni post test. A value of *P*<0.05 was considered to be statistically significant.

## Figures and Tables

**Table 1:** Sequences of primers used for RT-qPCR

**Gene**	**Sequences of primers**	**NCBI reference sequence**
*β*-Actin	F 5′-CATCCGTAAAGACCTCTATGCCAAC-3′	NM_007393.5
	R 5′-ATGGAGCCACCGATCCACA-3′	
TNF-*α*	F 5′-TCTTCTCATTCCTGCTTGTGG-3′	NM_001278601.1
	R 5′-GGTCTGGGCCATAGAACTGA-3′	
IL-6	F 5′-GGAGCCCACCAAGAACGATAG-3′	NM_001314054.1
	R 5′-GTGAAGTAGGGAAGGCCGTG-3′	
MCP-1	F 5′-GGCTCAGCCAGATGCAGTTAA-3′	NM_011333.3
	R 5′-CCTACTCATTGGGATCATCTTGCT-3′	
IL-1*β*	F 5′-TTGACGGACCCCAAAAGAT-3′	NM_008361.4
	R 5′-AGCTGGATGCTCTCATCAGG-3′	
iNOS	F 5′-CCCTTCAATGGTTGGTACATGG-3′	NM_001313922.1
	R 5′-ACATTGATCTCCGTGACAGCC-3′	
CD206	F 5′-TCTTTGCCTTTCCCAGTCTCC-3′	NM_008625.2
	R 5′-TGACACCCAGCGGAATTTC-3′	
Arg-1	F 5′-AGACAGCAGAGGAGGTGAAGAGTAC-3′	NM_007482.3
	R 5′-GGTAGTCAGTCCCTGGCTTATGGT-3′	
IL-10	F 5′-CAGAGCCACATGCTCCTAGA-3′	NM_010548.2
	R 5′-TGTCCAGCTGGTCCTTTGTT-3′	

Abbreviations: F, forward; R, reverse.

## Abbreviations

PD-1: Programmed cell death-1
MCP-1: Monocyte chemotactic protein-1
IL: Interleukin
LPM: Large peritoneal macrophages
SPM: Small peritoneal macrophages
BMDMs: Bone marrow–derived macrophages
TNF-*α*: Tumor necrosis factor-*α*
ELISA: Enzyme linked immunosorbent assay
iNOS: Inducible nitric oxide synthase
Arg-1: Arginase-1
AP-1: Activation protein-1
PI3K: phosphatidylinositide 3-kinases
PKB, Akt: Protein kinase B
STAT: Signal transducer and activator of transcription
JNK: c-Jun N-terminal kinase
NF-*κ*B: Nuclear factor kappa-light-chain-enhancer of activated B cells

## References

[bib1] Benoit M, Desnues B, Mege JL. Macrophage polarization in bacterial infections. J Immunol 2008; 181: 3733–3739. 18768823 10.4049/jimmunol.181.6.3733

[bib2] Gordon S, Martinez FO. Alternative activation of macrophages: mechanism and functions. Immunity 2010; 32: 593–604. 20510870 10.1016/j.immuni.2010.05.007

[bib3] Ghosna EE, Cassadoc AA, Govoni GR, Fukuhara T, Yang Y, Monack DM et al. Two physically, functionally, and developmentally distinct peritoneal macrophage subsets. Proc Natl Acad Sci USA 2010; 107: 2568–2573. 20133793 10.1073/pnas.0915000107 PMC2823920

[bib4] dos Anjos Cassado A, D'Império Lima MR, Bortoluci KR. Revisiting mouse peritoneal macrophages: heterogeneity, development, and function. Front Immunol 2015; 6: 225. 26042120 10.3389/fimmu.2015.00225 PMC4437037

[bib5] Okazaki T, Honjo T. The PD-1-PD-L pathway in immunological tolerance. Trends Immunol 2006; 27: 195–201. 16500147 10.1016/j.it.2006.02.001

[bib6] D-x Bu, Tarrio M, Maganto-Garcia E, Stavrakis G, Tajima G, Lederer J et al. Impairment of the programmed cell death-1 pathway increases atherosclerotic lesion development and inflammation. Arterioscler Thromb Vasc Biol 2011; 31: 1100–1107. 21393583 10.1161/ATVBAHA.111.224709 PMC3104026

[bib7] Brown KE, Freeman GJ, Wherry EJ, Sharpe AH. Role of PD-1 in regulating acute infections. Curr Opin Immunol 2010; 22: 397–401. 20427170 10.1016/j.coi.2010.03.007 PMC2905659

[bib8] Keir ME, Butte MJ, Freeman GJ, Sharpe AH. PD-1 and its ligands in tolerance and immunity. Annu Rev Immunol 2008; 26: 677. 18173375 10.1146/annurev.immunol.26.021607.090331 PMC10637733

[bib9] Takahashi M, Galligan C, Tessarollo L, Yoshimura T. Monocyte chemoattractant protein-1(MCP-1), not MCP-3, is the primary chemokine required for monocyte recruitment in mouse peritonitis induced with thioglycollate or zymosan A. J Immunol 2009; 183: 3463–3471. 19641140 10.4049/jimmunol.0802812 PMC7371094

[bib10] Taylor PR, Brown GD, Geldhof AB, Martinez-Pomares L, Gordon S. Pattern recognition receptors and differentiation antigens define murine myeloid cell heterogeneity *ex vivo*. Eur J Immunol 2003; 33: 2090–2097. 12884282 10.1002/eji.200324003

[bib11] Ansari MJ, Salama AD, Chitnis T, Smith RN, Yagita H, Akiba H et al. The programmed death-1 (PD-1) pathway regulates autoimmune diabetes in nonobese diabetic (NOD) mice. J Exp Med 2003; 198: 63–69. 12847137 10.1084/jem.20022125 PMC2196083

[bib12] Han MS, Jung DY, More C, Lakhani SA, Kim JK, Flavell RA et al. JNK expression by macrophages promotes obesity-induced insulin resistance and inflammation. Science 2013; 339: 218–222. 23223452 10.1126/science.1227568 PMC3835653

[bib13] Ma W, Gee K, Lim W, Chambers K, Angel JB, Kozlowski M et al. Dexamethasone inhibits IL-12p40 production in lipopolysaccharide-stimulated human monocytic cells by down-regulating the activity of c-Jun N-terminal kinase, the activation protein-1, and NF-kappa B transcription factors. J Immunol 2004; 172: 318–330. 14688340 10.4049/jimmunol.172.1.318

[bib14] Hae-Yun C, Eun-Kyoung C, Soo-Woon L, Jung KO, Seo SK, Choi IW et al. Programmed death-1 receptor negatively regulates LPS-mediated IL-12 production and differentiation of murine macrophage RAW264.7 cells. Immunol Lett 2009; 127: 39–47. 19723542 10.1016/j.imlet.2009.08.011

[bib15] Lawrence T, Natoli G. Transcriptional regulation of macrophage polarization: enabling diversity with identity. Nat Rev Immunol 2011; 11: 750–761. 22025054 10.1038/nri3088

[bib16] Sica A, Mantovani A. Macrophage plasticity and polarization: *in vivo* veritas. J Clin Invest 2012; 122: 787–795. 22378047 10.1172/JCI59643 PMC3287223

[bib17] Okazaki T, Maeda A, Nishimura H, Kurosaki T, Honjo T. PD-1 immunoreceptor inhibits B cell receptormediated signaling by recruiting src homology 2-domaincontaining tyrosine phosphatase 2 to phosphotyrosine. Proc Natl Acad Sci USA 2001; 98: 13866–13871. 11698646 10.1073/pnas.231486598 PMC61133

[bib18] Yao A, Liu F, Chen K. Programmed death 1 deficiency induces the polarization of macrophages/microglia to the M1 phenotype after spinal cord injury in mice. Neurotherapeutics 2014; 11: 636–650. 24853068 10.1007/s13311-013-0254-x PMC4121443

[bib19] Barth MW, Hendrzak JA, Melnicoff MJ, Morahan PS. Review of the macrophage disappearance reaction. J Leukoc Biol 1995; 57: 361–367. 7884305 10.1002/jlb.57.3.361

[bib20] Rosas M, Thomas B, Stacey M, Gordon S, Taylor. PR. The myeloid 7/4-antigen defines recently generated inflammatory macrophages and is synonymous with Ly-6B. J Leukoc Biol 2010; 88: 169–180. 20400676 10.1189/jlb.0809548 PMC2892525

[bib21] Biswas SK, Mantovani A. Macrophage plasticity and interaction with lymphocyte subsets: cancer as a paradigm. Nat Immunol 2010; 11: 889–896. 20856220 10.1038/ni.1937

[bib22] Riemann A, Wußling H, Loppnow H, Fu H, Reime S, Thews O. Acidosis differently modulates the inflammatory program in monocytes and macrophages. Biochim Biophys Acta 2015; 1862: 72–81. 26499398 10.1016/j.bbadis.2015.10.017

[bib23] Melgarejo E, Medina MÁ, Sánchez-Jiménez F, Urdiales JL. Monocyte chemoattractant protein-1: A key mediator in inflammatory processes. Int J Biochem Cell Biol 2009; 41: 998–1001. 18761421 10.1016/j.biocel.2008.07.018

[bib24] Said EA, Dupuy FP, Trautmann L, Zhang Y, Shi Y, El-Far M et al. Programmed death-1-induced interleukin-10 production by monocytes impairs CD4+ T cell activation during HIV infection. Nat Med 2010; 16: 452–459. 20208540 10.1038/nm.2106 PMC4229134

[bib25] Choi H, Lee RH, Bazhanov N, Oh JY, Prockop DJ. Anti-inflammatory protein TSG-6 secreted by activated MSCs attenuates zymosan-induced mouse peritonitis by decreasing TLR2/NF-*κ*B signaling in resident macrophages. Blood 2011; 118: 330–338. 21551236 10.1182/blood-2010-12-327353 PMC3138686

[bib26] Zhang TY, Daynes RA. Macrophages from 11beta-hydroxysteroid dehydrogenase type 1-deficient mice exhibit an increased sensitivity to lipopolysaccharide stimulation due to TGF-beta-mediated up-regulation of SHIP1 expression. J Immunol 2007; 179: 6325–6335. 17947710 10.4049/jimmunol.179.9.6325

[bib27] Li Q, Verma IM. NF-kappa B regulation in the immune system. Nat Rev Immunol 2002; 2: 725–734. 12360211 10.1038/nri910

[bib28] Ping D, Jones PL, Boss. JM. TNF regulates the *in vivo* occupancy of both distal and proximal regulatory regions of the MCP-1/JE gene. Immunity 1996; 4: 455–469. 8630731 10.1016/s1074-7613(00)80412-4

[bib29] Bally AP, Lu P, Tang Y, Austin JW, Scharer CD, Ahmed R et al. NF-kB regulates PD-1 expression in macrophages. J Immunol 2015; 194: 4545–4554. 25810391 10.4049/jimmunol.1402550 PMC4402259

[bib30] Keir ME, Freeman GJ, Sharpe AH. PD-1 regulates self-reactive CD8+ T cell responses to antigen in lymph nodes and tissues. J Immunol 2007; 179: 5064–5070. 17911591 10.4049/jimmunol.179.8.5064

[bib31] Chen W, Liu J, Meng J, Lu C, Li X, Wang E et al. Macrophage polarization induced by neuropeptide-methionine enkephalin (MENK) promotes tumoricidal responses. Cancer Immunol Immunother 2012; 61: 1755–1768. 22419372 10.1007/s00262-012-1240-6 PMC11028532

